# A Question of Ownership

**Published:** 1894-12

**Authors:** 


A QUESTION OF OWNERSHIP.—The *Provence Medicale* for October 13th, says: "Has a tooth that has just been extracted an owner, and, if it has, to whom does it belong? This singular question has been actually submitted to a court at Gera. A person who had suffered for a long time with violent toothache consulted a dentist who extracted a tooth. As it appeared to him to be an interesting specimen, he wished to preserve it and put it into a collection of curious things. The patient, however, claimed the tooth as his property, and, as an amicable agreement could not be established between them, the patient lodged a complaint of fraudulent appropriation against the dentist. The accused demanded the tooth and supported his claim by quoting ancient customs in similar cases. He maintained that when a tooth was extracted it became an object without an owner and that thus it fell into the possession of the dentist; consequently there could be no fraudulent appropriation or any punishable act."

